# Editorial: Neuroprotective mechanisms and translational approaches in the retina

**DOI:** 10.3389/fnins.2023.1140759

**Published:** 2023-01-26

**Authors:** Afsun Sahin, Markus Tschopp, Cavit Agca

**Affiliations:** ^1^Department of Ophthalmology, Koc University Medical School, İstanbul, Türkiye; ^2^Department of Ophthalmology, Cantonal Hospital Aarau, Aarau, Switzerland; ^3^Department of Ophthalmology, Inselspital Universitatsspital Bern, Bern, Switzerland; ^4^Molecular Biology, Genetics and Bioengineering Program, Sabanci University, İstanbul, Türkiye; ^5^Nanotechnology Research and Application Center (SUNUM), Sabanci University, İstanbul, Türkiye

**Keywords:** neuroprotection, gene therapy, CRISPR-Cas9, hypoxia, retinal metabolism

Many blinding diseases including age-related macular degeneration, diabetic retinopathy, glaucoma, and inherited retinal diseases (IRDs) lead to retinal neurodegeneration and subsequent visual impairment. Despite the recent advancements in medicine, there is only a limited number of therapies and drugs available to stop the ongoing degeneration during these conditions. The current special topic emphasizes the importance of neuroprotective approaches and covers original research articles and reviews on both *in vivo* and *in vitro* applications and several different concepts including hypoxia, retinal detachment, diabetic retinopathy, glaucoma, autophagy, gene editing, and gene regulatory therapies in the retina. The special topic also includes reviews on *Retbindin* (*Rtbdn*) and *Thioredoxin*-*interacting protein* (*TXNIP*) as potential therapeutic targets for future neuroprotective approaches.

Retinal detachment is a serious condition, which may eventually lead to visual impairment. Chidlow et al. investigated the photoreceptor metabolism during retinal detachment. The authors used an experimental animal model for retinal detachment and showed the downregulation of both glycolytic and mitochondrial enzymes during detachment, which suggested a reduction in photoreceptor metabolism. Interestingly, the lack of oxygen availability or nutrient supply in the experimental detachment model did not lead to HIF-1α nuclear localization or pimonidazole labeling. Furthermore, targeting the metabolic pathways through pyruvate supplementation did not yield a neuroprotective effect. These findings already advanced our basic understanding of retinal detachment and will contribute to further neuroprotective approaches.

Short-term hypoxic stress and the related well-known factors HIF-1α and HIF-2α have been the subject of numerous neuroprotective approaches in retinal disease models. Stanniocalcin-1 (STC1) and Stanniocalcin-2 (STC2) are both regulated by hypoxic conditions and STC1 has been shown to be neuroprotective during photoreceptor degeneration. Ail et al. studied the expression profile of *Stc1* and *Stc2* in both hypoxic and degenerating retinas. By using transgenic animal models, authors also showed that *Stc2* is expressed in a HIF-1α dependent manner. However, the absence of *Stc1* or *Stc2* did not induce a profound effect on healthy retinas. Ail et al. demonstrated key findings for the elucidation of the neuroprotective potential of *Stc1* and *Stc2*. Further studies using the *Stc1* and *Stc2* transgenic models will also help to better understand the neuroprotective effects of these two factors.

The role of autophagy in glaucomatous optic neuropathy and the related neuroprotective approaches targeting the autophagy pathways are very intriguing subjects. Ishikawa et al. used autophagy-modulating drugs to be able to show the involvement of autophagy in *in vivo* ocular hypertension (OHT) model. The authors also showed that both AlloP and the enantiomeric AlloP (nt-AlloP) have neuroprotective effects in the OHT model of glaucoma in rats. Moreover, the neuroprotective effects of nt-AlloP in OHT are hampered by the autophagy inhibitor, 3-MA, suggesting that the neuroprotective effects of nt-AlloP mainly depend on its autophagy-modulating effect. This study clearly showed the interplay between OHT and autophagy and defined autophagy as a critical component in the OHT model of glaucoma.

Mohamad et al. also studied the neuroprotective factors for retinal ganglion cells (RGCs) using NMDA-induced excitotoxicity which is a well-known model for RGC loss. The authors showed that the neuroprotective effects of PhTX-343 suppressed the NMDA-induced defects in retinal morphology and function. This study elucidated the effects of another important neuroprotective factor for RGC survival.

Metabolic dysregulation in retinal disease is one of the emerging topics in IRD studies. *Rtbdn*, is a regulator of retinal hemostasis through Flavin metabolism and also has important roles as a neuroprotective factor. The absence of *Rtbdn* accelerates the disease outcome in several disease models. Zhao et al. gave a detailed summary of *Rtbdn*‘s role in IRD disease models and underline its potential therapeutic usage as a general neuroprotective approach for different IRDs.

Yet, another potential therapeutic target for retinal neuroprotection is the *TXNIP* which is known to be an important factor for diabetic retinopathy condition. Liu et al. summarized the function of TXNIP and its role in diabetic retinopathy. The authors also reviewed the potential benefits of inhibiting TXNIP in diabetic retinal neurodegeneration (DRN) and overviewed the animal models that alleviate DRN.

*In vitro* methods and technologies are also important to test and evaluate neuroprotective therapies for retinal diseases. In that sense, Zhu et al. provided an in-depth review of *in vitro* methods including retinal organoids, organotypic retinal explants, and whole eye cultures. The authors also summarized the available retinal cell lines and the approaches and compared their advantages and limitations in detail. They also reviewed *in vitro* neuroprotective studies using different pharmacological agents. This was particularly important to test and evaluate the drug of interest, reduce animal numbers, and also to develop drug delivery systems with the help of suitable *in vitro* methodology.

Finally, all the recent proof-of-concept and clinical studies for neuroprotective gene therapy applications have been reviewed by Altay et al.. Meta-analysis of the ongoing clinical trials showed that ophthalmology is one of the leading fields in gene therapy together with cancer, hematology, and the diseases of the nervous system. Further analysis also showed that AAV vectors dominate the existing gene therapies in the ophthalmology field. Altay et al. also provided an in-depth review of CRISPR-Cas systems, like base editing, prime editing, and CasRx in retinal gene therapy applications. Further gene regulatory approaches including the dCas9, Zinc finger or TALE-based artificial transcription factors, and the antisense oligonucleotides for the retinal disease are summarized. The authors also reviewed a novel protein delivery method for a Zinc-finger based artificial transcription factor.

Overall, this special topic covered several retinal studies and reviews for neuroprotective approaches and provided a valuable and timely resource for future neuroprotective applications for degenerating retinal diseases.

## Author contributions

The editorial was written by CA and MT. The editorial was edited by CA, MT, and AS. The article's submission was reviewed and approved by all authors.

